# Successful laparoscopic resection for cap polyposis: case report, literature review

**DOI:** 10.1186/s40792-018-0476-6

**Published:** 2018-07-03

**Authors:** Koichi Tamura, Kenji Matsuda, Shozo Yokoyama, Hiromitsu Iwamoto, Yuki Mizumoto, Yuki Nakamura, Daisuke Murakami, Hiroki Yamaue

**Affiliations:** Second Department of Surgery, School of Medicine, Wakayama Medical University, 811-1, Kimiidera, Wakayama, 641-8510 Japan

**Keywords:** Cap polyposis, Hypoproteinemia, Laparoscopic surgery

## Abstract

**Background:**

Cap polyposis is a rare gastrointestinal disease with endoscopically and pathologically distinctive features. Its exact etiology has not been fully elucidated. In a few cases, there was recurrence after inadequate treatment. Efficacy of *Helicobacter pylori* eradication therapy, however, has been shown in some published research.

**Case presentation:**

A 70-year-old female patient developed intermittent mucous diarrhea with loss of body weight and visited a physician. Total colonoscopy showed multiple sessile polyps which were partially coadunated from the rectum to the sigmoid colon. Histopathological finding was tubular adenoma with mild atypia. The patient stayed for observation. Worsening symptoms following protein-losing enteropathy demanded surgical treatment because malignancy could not be ruled out. Laparoscopic resection was performed, and the surgical specimens revealed that the polypoid lesion mainly consisted of mild adenomatous glands which were covered with purulent granulation tissues. We made final diagnosis of cap polyposis and saw rapid improvement of her symptoms. Long-term observation is required after surgery.

**Conclusions:**

We reported a case of successful laparoscopic resection of cap polyposis with protein-losing enteropathy (170 words).

## Background

Cap polyposis is a rare benign entity. It is typically characterized by inflammatory sessile polyps which are covered with ‘caps’ of purulent granulation tissue. Typical symptoms are mucous diarrhea and rectal bleeding, which result in protein and body weight loss. Habitual straining during defecation and chronic constipation may also contribute to this disorder. Specific treatment has not been fully established, and since Williams et al. reported the first cases in 1985 [1], etiology has remained unknown. Several reports have shown the clinical courses and surgical treatments for this disease, including some recurring cases [2–8]. Here, we present a case of successful laparoscopic resection of cap polyposis and review literature about the surgical interventions for this disease.

## Case presentation

**Fig. 1:**
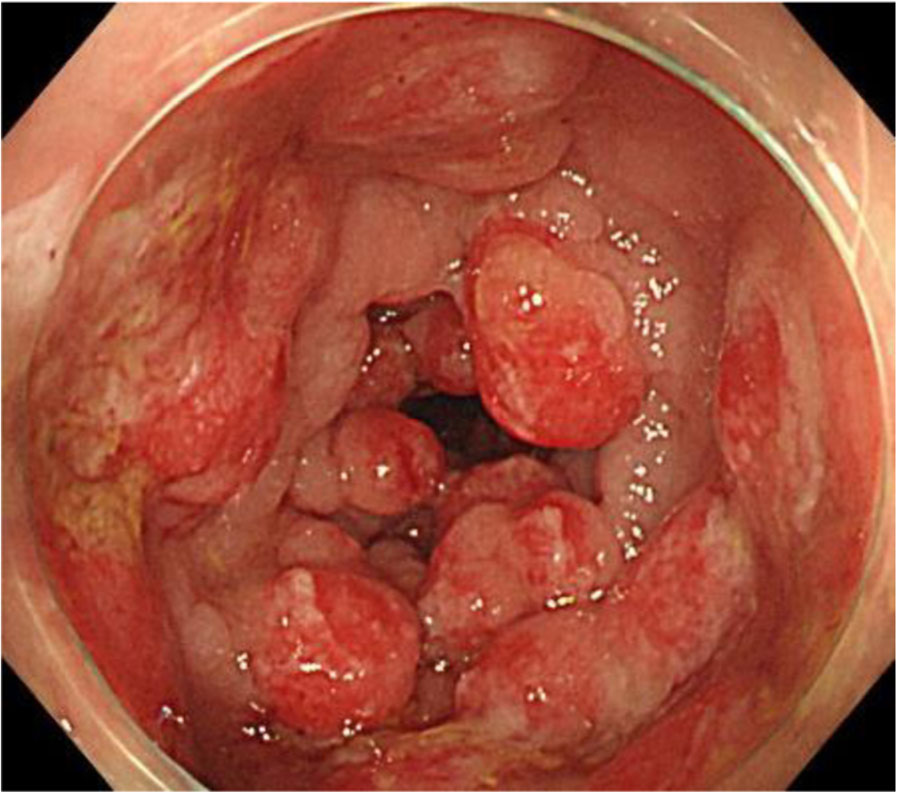
Colonoscopy showed multiple reddish polyps from the rectum to the sigmoid colon. Intervening mucosa showed no inflammatory change

**Fig. 2:**
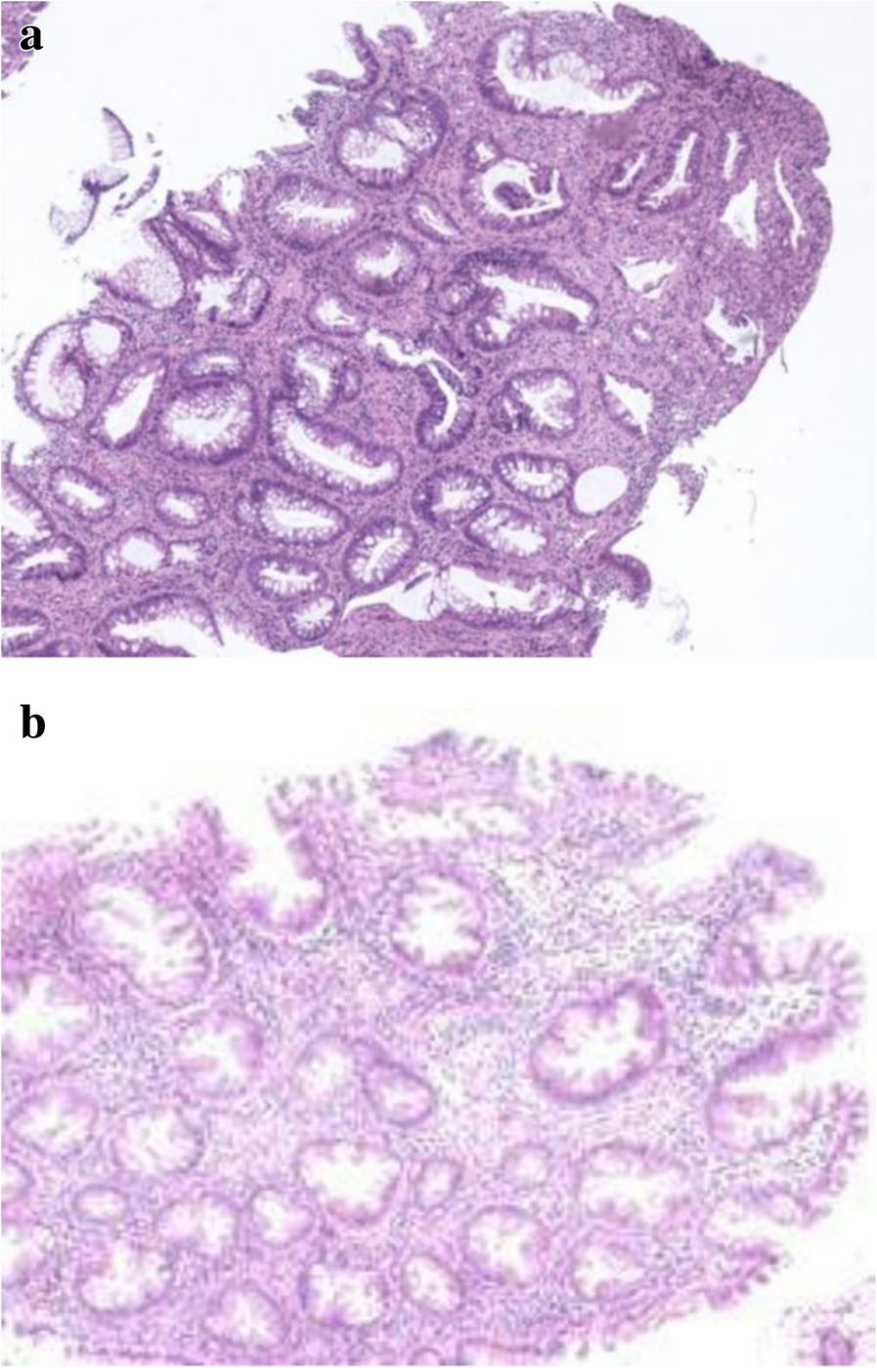
Histopathological sections of biopsy specimens revealed mild adenomatous glands (A) and serrated glands with no atypia that was the architectural feature of hyperplastic polyp (B). (× 40 magnification, Hematoxylin Eosin staining)

A 70-year-old woman visited another clinic with loss of body weight and mucous diarrhea. She had no family history and no characteristic travel history. She underwent total colonoscopy, which showed erythematous polypoid lesions from the lower rectum to the sigmoid colon (Fig. 1). Biopsy was taken from several typical polyps. Histopathological examination of biopsy specimens revealed hyperplastic polyps or tubular adenomas with low-grade atypia (Fig. 2). She was not judged to be an appropriate case for complete endoscopic polypectomy because of multiple polypoid lesions. She underwent gastroscopy, where slight atrophic gastritis with erythematous edema was confirmed. She was not diagnosed with *Helicobacter pylori* (*H. pylori*) infection by rapid urease tests and histopathological findings.

6 months later, the patient returned to clinic with a worse complaint of repeated mucous defecation (5–6 times/day) and bilateral leg edema. Second colonoscopy revealed no remarkable changes of polypoid lesions with purulent mucus. Biopsy specimens showed tubular adenomas which contained moderate atypia. Laboratory tests showed hypoproteinemia (serum total protein 6.0 g/dl; normal range 6.6–8.1 g/dl) and hypoalbuminemia (serum albumin 3.3 g/dl; normal range 4.1–5.1 g/dl) while white blood cell count (5800/mm^3^) and C-reactive protein levels (0.28 mg/dl) were not elevated. Other laboratory data were unremarkable. The patient sometimes developed tenesmus and mucous bloody diarrhea, and visited our hospital for detailed treatments.

**Fig. 3:**
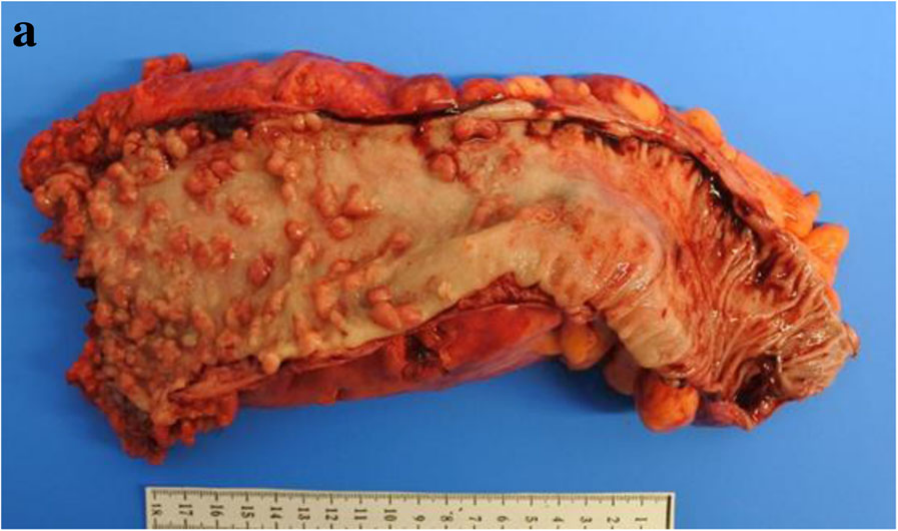
Surgical specimen showed more than 100 polyps from the rectum to the sigmoid colon

**Fig. 4:**
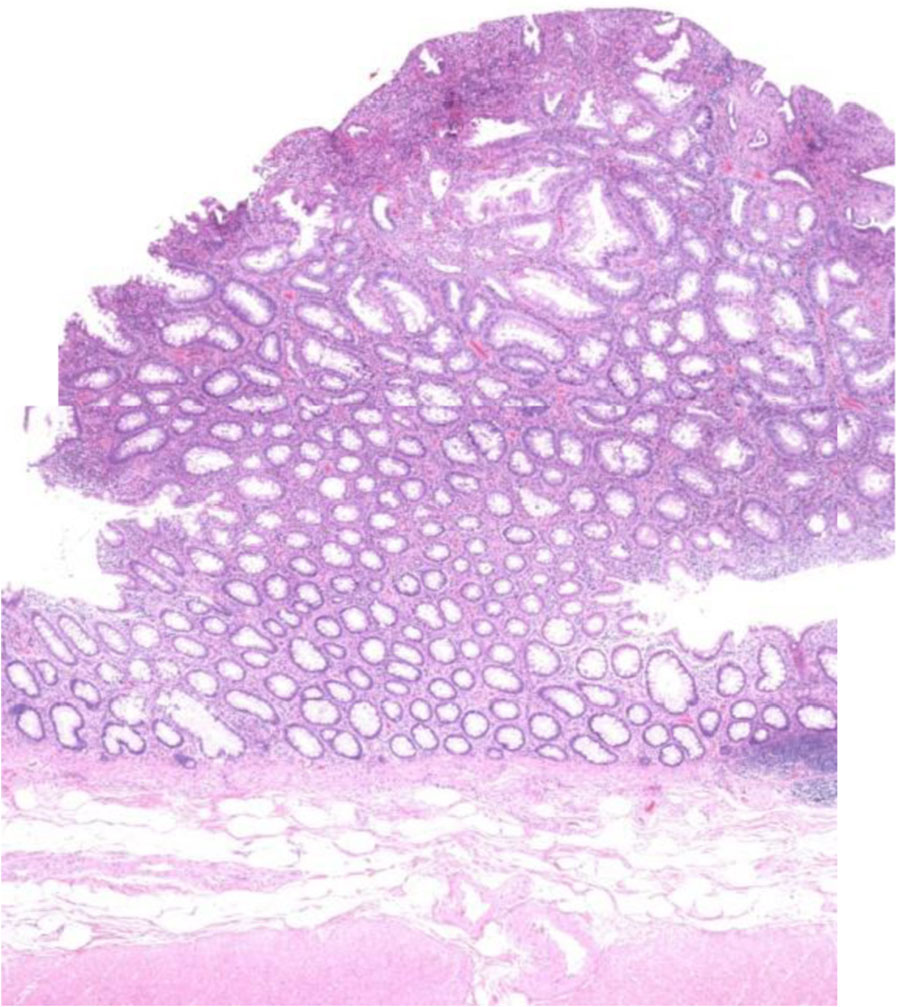
Histopathological findings were mild adenomatous glands with mixed inflammatory infiltration. The polyp was covered with inflammatory granulation tissue, a ‘cap.’ (× 40 magnification, Hematoxylin Eosin staining)

Considering the overall course of examination and taking into account the patient’s strong request for surgery, we judged she was not suffering from inflammatory bowel diseases or hereditary/non-hereditary polyposis coli and diagnosed protein-losing enteropathy which could not be denied malignant potential. Therefore, we performed laparoscopic low anterior resection of the rectum and the sigmoid colon with diverting ileostomy. During operation, we found thick subserosal layer with moderate fibrosis and edematous change. Anal surgical margin was located 3 cm above the dentate line. Surgical specimen revealed more than 100 polyps consisting of elongated and mild adenomatous glands with a thick layer of subserosal tissue (Fig. 3). Polyps were covered with a layer of inflammatory granulation tissues and fibrinopurulent exudate. The intervening mucosa of the polyps was relatively normal, so the patient was diagnosed with cap polyposis (Fig. 4). After surgery, symptoms improved, including return to normal range of serum total protein and albumin levels. 6 months later, she had no complaints or recurrence of cap polyposis in the remnant colon. We will continue a tight follow-up surveillance.

## Discussion

Cap polyposis was first reported by Williams et al. in 1985 [1]. Histopathological features are multiple sessile polypoid lesions that consist of serpentine and elongated glands with inflammatory change, its surface is covered with a ‘cap’ of fibrinopurulent exudates and granulation tissues. These polyps often affect the area between the rectum and the sigmoid colon. Previous literature showed that polypoid lesions were mainly associated with mucosal prolapse syndrome, fibromuscular obliteration, and chronic straining at defecation. Also, its rarity often made physicians diagnose ulcerative colitis or another inflammatory bowel disease with pseudopolyps [3, 6, 8, 9]. The exact etiology of cap polyposis remains unknown and several medical treatments, including aminosalicylates, antibiotics, steroids, and anti-inflammatory agents, have been recommended. In 2002, Oiya et al. showed complete remission of cap polyposis by administration of *H. pylori* eradication therapy [10]. Based on this report, patients diagnosed with cap polyposis following any uncontrollable symptoms are recommended treatment by *H. pylori* eradication therapy first.

We examined cases of cap polyposis received surgical interventions from a PubMed search of the English literature since 2002, when *H. pylori* eradication therapy was first reported (Table 1). Twelve cases of surgically treated cap polyposis were reported between 2004 and 2013, although several cases might have been experienced before 2002. In this reviewed series, last four cases [5–8] could not get the preoperative diagnosis of cap polyposis, and no case was confirmed which *H. pylori* was infected or not, and received *H. pylori* eradication therapy before any surgical treatment. One case underwent sigmoidectomy for cap polyposis and recurred after surgery [3]. She was diagnosed with *H. pylori* infection after screening examination and received *H. pylori* eradication therapy. There was no recurrence of cap polyposis. Further long-term reports of this disease are required to ascertain the appropriate strategy for diagnosis and for the optimal timing of *H. pylori* eradication therapy.

**Table 1:** Cases of cap polyposis treated by surgery from the previously published English literature

No.	Author	Age (years)	Gender	Initial symptoms	Preoperative treatment	Surgical procedure	Postoperative outcome
1	Ng et al. [2], 2004^a^	20	M	Unknown	Unknown	Transanal resection	Recurred at 3 months
2	Ng et al. [2], 2004^a^	15	M	Unknown	Unknown	Anterior resection	Resolved
3	Ng et al. [2], 2004^a^	21	M	Unknown	Unknown	Low anterior resection	Resolved
4	Ng et al. [2], 2004^a^	16	M	Unknown	Unknown	Perineal proctectomy	Recurred at 1 months
5	Ng et al. [2], 2004^a^	18	M	Unknown	Complete polypectomy	Anterior resection	Resolved
6	Ng et al. [2], 2004^a^	20	M	Unknown	Complete polypectomy	Anterior resection	Resolved
7	Akamatsu et al. [3], 2004	50	F	Mucous bloody stool	None	Sigmoidectomy	Recurred at 6 months
8	Konishi et al. [4], 2005	76	F	None	None	Sigmoidectomy	Worsen at anastomotic line
9	Gallegos et al. [5], 2011	56	M	Diarrhea, leg edema	Metronidazole	Left hemicolectomy	Recurred at 8 months
10	Kini et al. [6], 2012	19	M	Diarrhea, body weight loss	Steroids and anti- inflammatory agents^b^	Proctocolectomy	Resolved
11	Mason et al. [7], 2013	42	M	Bowel obstruction	None	Left hemicolectomy	Recurred at 1 year
12	Aggarwal et al. [8], 2013	27	M	Anemia	Aminosalicylates and steroids^c^	Low anterior resection	Resolved
13	Present case	70	F	Body weight loss, mucous diarrhea	None	Laparoscopic low anterior resection	Resolved

^a^Previous cases reviewed

^b^Initial diagnosis was Crohn’s disease

^c^Initial diagnosis was ulcerative colitis

In spite of three endoscopic biopsy examinations, our case could not be diagnosed as having cap polyposis. The patient also had no *H. pylori* infection. Acute exacerbation of symptoms at defecation might eventually urge her to receive surgical intervention to improve quality of life. After laparoscopic surgery, surgical specimens showed a slightly atypical feature of cap polyposis. Specifically, most polyps contained tubular adenoma structures with mild atypia and scattered hyperplastic polyps. These histopathological characteristics made determination of the appropriate treatment difficult. We might postoperatively perform *H. pylori* eradication therapy into account, if she got the definitive diagnosis of cap polyposis with *H. pylori* infection.

## Conclusions

We herein demonstrated a surgically treated case of cap polyposis with severe symptoms, which were unable to be preoperatively confirmed by repeated biopsy. As this disease shows various symptoms and endoscopically distinctive features, careful medical history taking and whole or adequate biopsy should be conducted for exact diagnosis. Our case review showed that it is difficult to confirm the diagnosis of cap polyposis and the necessity of strict surveillance for recurrence of the remnant colon.
